# Treatment Approaches for Psychiatric and Cognitive Comorbidities in Pediatric Epilepsy: A Systematic Review of Clinical Trials

**DOI:** 10.7759/cureus.85300

**Published:** 2025-06-03

**Authors:** Airin Parvin Nipu, Vinay Gundareddy, Irum Raza, Faizan Hassan, Muhammad Mairaj, Zain Ullah, Kinza Binte Khalid, Diego Jiménez Royg, Mofiyinfoluwa O Oloba, Mmahaletchumy Manoharan, Sajid Ali

**Affiliations:** 1 Internal Medicine, Mymensingh Medical College, Mymensingh, BGD; 2 Internal Medicine, Nathan Kline Institute for Psychiatric Research, Lagrangeville, USA; 3 Internal Medicine, Shalamar Medical and Dental College, Lahore, PAK; 4 Urology, Rehman Foundation, Sialkot, PAK; 5 Pediatrics, Islam Medical and Dental College, Sialkot, PAK; 6 Medicine and Surgery, Chandka Medical College, Larkana, PAK; 7 Internal Medicine, Bolan University of Medical and Health Sciences, Quetta, PAK; 8 Pediatrics, Liaquat National Hospital and Medical College, Karachi, PAK; 9 Internal Medicine, National University of Asunción, Asunción, PRY; 10 Pediatrics, Shandong University, Jinan, CHN; 11 Surgery, University of North Sumatra, Medan, IDN; 12 Cardiology, Rawalpindi Institute of Cardiology, Rawalpindi, PAK

**Keywords:** adhd, anxiety, cognitive behavioral therapy, cognitive impairment, epilepsy comorbidities, pediatric epilepsy, psychiatric symptoms, psychoeducation, psychological interventions, quality of life

## Abstract

This systematic review examines the effectiveness of treatment strategies aimed at addressing psychiatric symptoms and cognitive impairments in children with epilepsy. Pediatric epilepsy is commonly accompanied by mental health challenges such as anxiety, depression, attention difficulties, and problems with executive functioning, all of which can significantly affect quality of life. A thorough search of recent clinical trials was conducted to evaluate both pharmacological and non-pharmacological interventions. Ten studies involving 879 participants between the ages of three and 19 were included. The interventions reviewed encompassed psychological therapies, educational support programs, dietary supplements, and digital tools. Several interventions demonstrated notable improvements in emotional regulation, behavioral functioning, and family dynamics. The evidence consistently highlights the benefits of integrated, family-oriented approaches in enhancing cognitive and psychological outcomes in this population. Despite encouraging results, limitations such as small sample sizes, short follow-up periods, and variability in study designs indicate a need for further high-quality research. These findings emphasize the importance of incorporating mental health and cognitive support as essential elements of pediatric epilepsy care and call for greater efforts to develop standardized, accessible treatment models.

## Introduction and background

Children with epilepsy frequently experience not only recurrent seizures but also a wide range of neuropsychiatric and cognitive challenges that profoundly impact their quality of life. Epilepsy is one of the most common chronic neurological conditions in childhood, with an estimated prevalence of three to five per 1,000 children globally [1,2]. Beyond seizure control, a growing body of literature emphasizes that cognitive impairments, including deficits in attention, memory, executive functioning, and language development, are prevalent in pediatric epilepsy populations [3]. These cognitive deficits can be exacerbated by both the underlying neurological etiology and the epileptic activity itself. Moreover, psychiatric comorbidities such as anxiety, depression, attention-deficit/hyperactivity disorder (ADHD), and conduct disorders are significantly more common in children with epilepsy than in their healthy peers, with some studies reporting rates as high as 50-60% [4].

The interplay between epilepsy, cognition, and psychiatric symptoms is complex and multifactorial. Several contributing factors have been proposed, including seizure frequency, age at onset, underlying structural brain abnormalities, effects of antiseizure medications, and psychosocial stressors [5]. In some cases, the cognitive and psychiatric symptoms may precede the diagnosis of epilepsy or persist even when seizures are well-controlled, highlighting the need for comprehensive long-term care. These impairments can interfere with academic performance, social integration, and emotional development, resulting in significant burdens on affected children and their families. Consequently, the timely identification and management of psychiatric and cognitive issues have become critical components of holistic epilepsy care [6].

If left unaddressed, psychiatric and cognitive comorbidities in pediatric epilepsy can contribute to long-term developmental delays, reduced medication adherence, increased healthcare utilization, and poorer educational and psychosocial outcomes. The emotional and financial strain on caregivers may also be amplified, compounding the overall burden of disease. Therefore, early and sustained intervention is essential not only for improving individual outcomes but also for mitigating broader societal impacts.

A variety of treatment approaches have been developed and investigated to address these concerns. Pharmacologic interventions such as the use of selective serotonin reuptake inhibitors (SSRIs), antipsychotics, or stimulants have shown promise in managing psychiatric comorbidities, although their use in pediatric epilepsy requires caution due to potential interactions with antiseizure drugs and the risk of seizure exacerbation [7]. Non-pharmacologic strategies, including cognitive behavioral therapy (CBT), psychoeducational programs, parent-led interventions, and school-based support systems, have gained traction as safer and often more sustainable alternatives [8]. Additionally, nutritional supplementation (e.g., omega-3 fatty acids), dietary therapies, and neuromodulatory treatments have been explored for their effects on both seizure control and cognitive or emotional regulation. However, the evidence remains scattered, and comparative effectiveness across these diverse modalities is still under active investigation.

Given the significant and multifaceted burden of psychiatric and cognitive impairments in children with epilepsy, this systematic review aims to synthesize and evaluate existing evidence on treatment strategies targeting these comorbidities. The review will address the Population, Intervention, Comparison, and Outcome (PICO) question [9]: In children diagnosed with epilepsy (Population), how do various pharmacological, psychological, and supportive interventions (Intervention), compared to standard epilepsy care or no adjunctive treatment (Comparison), affect psychiatric symptoms and cognitive outcomes (Outcome)? The PICO framework was chosen to provide a structured and transparent method for defining the clinical question, guiding inclusion criteria, and ensuring a focused synthesis of the most relevant and high-quality evidence to inform clinical decision-making.

Unlike previous reviews that have largely centered on seizure control or isolated treatment modalities, this review offers a broader and more integrated perspective by including recent clinical trials that assess both pharmacological and non-pharmacological strategies aimed specifically at improving cognitive and psychiatric outcomes. This approach helps bridge an important gap in the literature and underscores the need for comprehensive, multimodal management strategies in pediatric epilepsy.

## Review

Materials and methods

Search Strategy

The search strategy for this systematic review was developed in accordance with the Preferred Reporting Items for Systematic Reviews and Meta-Analyses (PRISMA) guidelines [10] to ensure methodological transparency and reproducibility. A comprehensive electronic search was conducted across four major biomedical databases: PubMed, Scopus, Embase, and the Cochrane Central Register of Controlled Trials. Search terms included both controlled vocabulary (e.g., Medical Subject Headings (MeSH) terms) and free-text keywords such as “epilepsy,” “children,” “cognitive impairment,” “psychiatric symptoms,” “behavioral issues,” and “interventions,” combined using Boolean operators (AND, OR) to optimize sensitivity and specificity.

To ensure the inclusion of the most current and clinically relevant evidence, the search was restricted to peer-reviewed clinical trials published in English within the last five years (2019-2024). This time frame was selected to reflect recent advances in treatment modalities, including digital health tools, updated therapeutic guidelines, and the growing emphasis on psychosocial interventions in pediatric epilepsy care. Older studies were excluded to maintain the review’s focus on evidence applicable to contemporary clinical practice.

Study screening and selection were conducted independently by two reviewers. Disagreements regarding inclusion criteria or data extraction were resolved through discussion; a third reviewer was involved when consensus could not be reached. Although the protocol for this review was not registered on the International Prospective Register of Systematic Reviews (PROSPERO), the methodology followed standard systematic review procedures to maintain rigor, transparency, and replicability.

Eligibility Criteria

Studies were included in this review based on predefined eligibility criteria aligned with the study's objective of evaluating treatment approaches for psychiatric symptoms and cognitive impairments in children with epilepsy. Eligible studies were required to be clinical trials involving participants aged three to 19 years diagnosed with epilepsy and reporting on at least one psychiatric or cognitive outcome, such as anxiety, depression, attention deficits, executive function, or quality of life. Only studies published in English within the last five years were considered to ensure relevance and recency. Both pharmacological and non-pharmacological interventions were eligible for inclusion, while studies involving adults, non-interventional designs, editorials, case reports, or non-peer-reviewed sources were excluded. This criterion ensured a focused selection of high-quality, evidence-based trials pertinent to the pediatric epilepsy population.

Data Extraction

A standardized data extraction form was developed and used to systematically collect relevant information from each included study. Key variables extracted included the study title and author, publication year, study design, sample size, age range or mean age of participants, type of epilepsy or comorbid diagnosis, type of intervention and comparator, psychiatric and/or cognitive outcomes assessed, and key findings. Extraction was conducted independently by two reviewers to ensure accuracy and minimize bias, with discrepancies resolved through discussion and consensus. This structured process ensured consistent documentation of clinical and methodological characteristics across all studies and supported a robust comparative synthesis of findings.

Data Analysis and Synthesis

Given the heterogeneity in study designs, intervention types, and outcome measures, a narrative synthesis approach was used in place of a meta-analysis. The results were organized based on intervention categories: psychological, educational, pharmacological, or dietary, and analyzed for their reported effects on psychiatric and cognitive domains such as behavior, attention, emotional regulation, and quality of life. The synthesis aimed to identify consistent patterns, highlight variations across studies, and pinpoint gaps in the existing literature. Where applicable, the methodological quality and risk of bias of each included study were assessed using standardized tools. These included the Risk of Bias 2.0 (RoB 2.0) tool developed by the Cochrane Collaboration, London, UK [11], the Critical Appraisal Skills Programme (CASP) checklist created by the Oxford Centre for Triple Value Healthcare, Oxford, UK [12], and the Mixed Methods Appraisal Tool (MMAT) developed by researchers at McGill University, Montréal, Canada [13] for appraising mixed-methods studies. This structured approach supported a comprehensive and nuanced interpretation of the complex data related to pediatric epilepsy care.

Results

Study Selection Process

**Figure 1:**
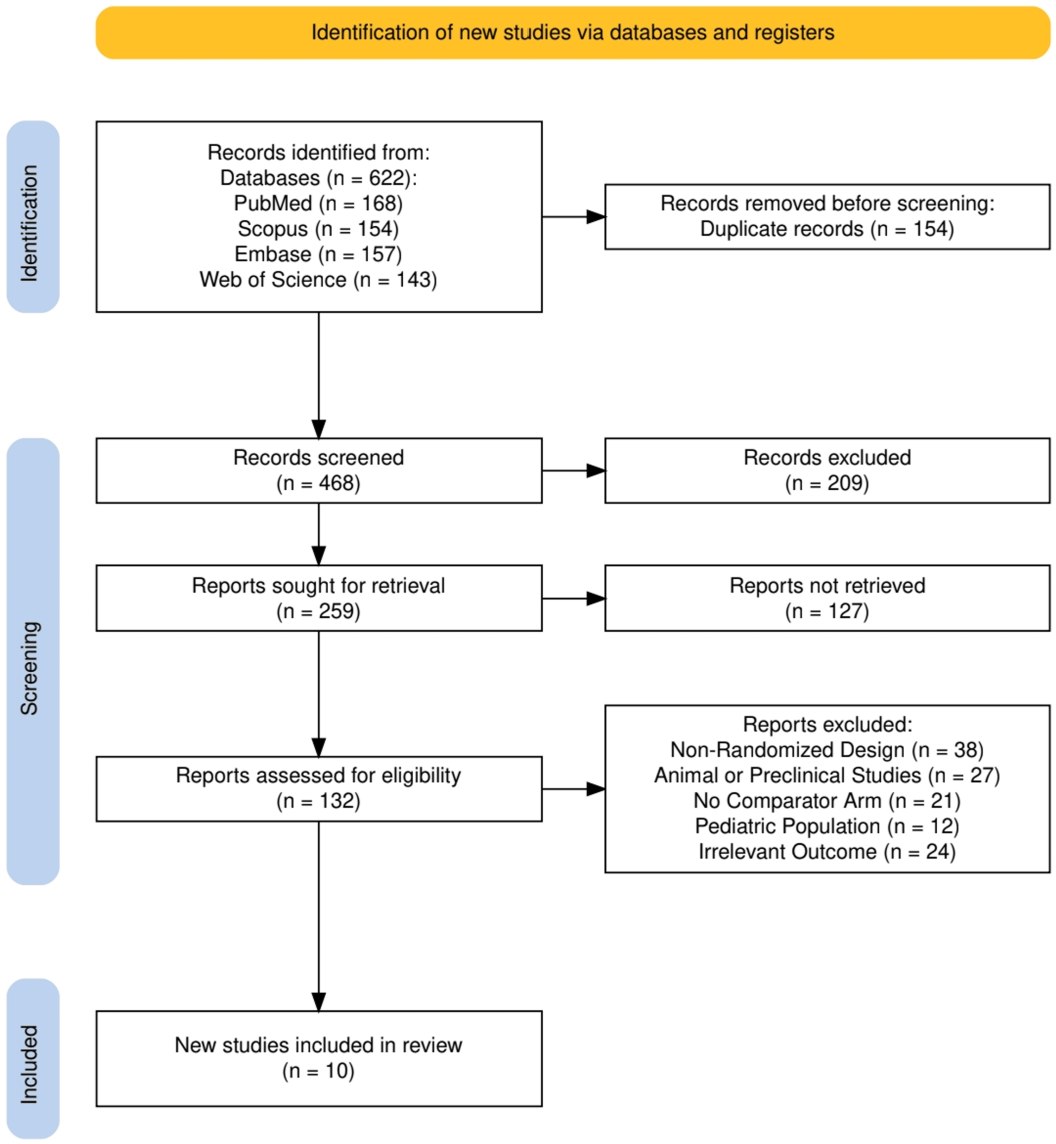
The PRISMA flowchart represents the study selection process. PRISMA: Preferred Reporting Items for Systematic reviews and Meta-Analyses

The study selection process is illustrated in Figure 1, following the PRISMA 2020 flow diagram. A total of 622 records were identified through database searches, including PubMed (n = 168), Scopus (n = 154), Embase (n = 157), and Web of Science (n = 143). After removing 154 duplicate records, 468 records were screened based on titles and abstracts. Of these, 209 were excluded for not meeting basic inclusion criteria. Full-text retrieval was sought for 259 reports, of which 127 could not be retrieved. A total of 132 full-text articles were assessed for eligibility. Of these, 122 were excluded due to reasons including non-randomized design (n = 38), animal or preclinical studies (n = 27), absence of a comparator arm (n = 21), non-pediatric population (n = 12), or irrelevant outcome measures (n = 24). Ultimately, 10 studies met the inclusion criteria and were incorporated into the final qualitative synthesis.

Characteristics of the Selected Studies

The characteristics of the 10 studies included in this review are summarized in Table 1. All studies were published within the last five years and focused on pediatric populations with epilepsy, with participant age ranges spanning from three to 19 years. A majority were randomized controlled trials, including both open-label and double-blind designs, while others employed mixed-methods or qualitative approaches embedded within larger trials. The interventions assessed encompassed both pharmacological and non-pharmacological strategies, including structured psychological therapies, nurse-led CBT, psychoeducational programs, dietary supplementation, and web-based education platforms. The targeted psychiatric and cognitive outcomes varied across studies, with common assessments focusing on emotional and behavioral symptoms, executive functioning, attention, quality of life, and parental understanding or anxiety. Notably, several studies addressed comorbidities such as ADHD, ASD, or ID, reflecting the diverse clinical presentations within the pediatric epilepsy population. Most interventions demonstrated favorable outcomes, especially those involving family-centered psychological and educational components, highlighting their value in comprehensive epilepsy care.

**Table 1:** Summary of clinical trials assessing treatment strategies for psychiatric and cognitive comorbidities in pediatric epilepsy. ASD: autism spectrum disorder; CBT: cognitive behavioral therapy; ID: intellectual disability; MICE: Mental Health Intervention for Children With Epilepsy; SDQ: Strengths and Difficulties Questionnaire; SPACE: supporting attention in children with epilepsy; HRQOL: health-related quality of life; ADHD: attention-deficit/hyperactivity disorder; CBD: cannabidiol; AEDs: antiepileptic drugs; WEEP: Web-based Epilepsy Education Program; NIHTB-CB: NIH Toolbox Cognition Battery; ABAS-II: Adaptive Behavior Assessment System–Second Edition

Study (Author, Year)	Study Design	Sample Size	Age Range/Mean Age	Type of Epilepsy/Diagnosis	Intervention	Comparator	Psychiatric/Cognitive Outcomes Assessed	Key Findings
Bennett et al., 2024 [14]	Multicentre RCT (open-label)	334 randomized	3–18 years (mean ~10.5)	Epilepsy with mental health disorders	MICE + usual care	Assessment-enhanced usual care	SDQ total difficulties score	Improved SDQ scores in the MICE group; fewer adverse events
Nizza et al., 2024 [15]	Mixed-methods (within RCT)	25	3–18 years	Epilepsy with mental health difficulties	MICE	Not applicable	Emotional regulation, behavior, and parental understanding	Improved emotion regulation and behavioral awareness
Smith et al., 2025 [16]	Qualitative study within RCT	24 families	3–18 years	Epilepsy with mental health difficulties (ASD, ID)	MICE	Not applicable	Parent–child relationship, parental self-perception	Improved parental understanding and relationships
Wu et al., 2024 [17]	RCT	238	Not stated	Children with epilepsy	Nurse-led CBT for parents	Standard discharge care	Parental anxiety/depression, sleep, attitudes	Significant improvements in mental health and satisfaction
Svanström et al., 2023 [18]	Single-arm pilot trial	16	8–13 years	Epilepsy with attention difficulties	SPACE psychoeducational program	None	Executive function, HRQOL, attention	Significant improvements in executive function and HRQOL
Thompson et al., 2020 [19]	Open-label clinical trial	38	3–19 years	Treatment-resistant epilepsy	CBD (Epidiolex®)	None	NIHTB-CB, ABAS-II	No adverse effects; non-significant trend toward cognitive improvement
Rheims et al., 2024 [20]	RCT (placebo-controlled)	74	>6 to <16 years	Epilepsy with ADHD	PS-Omega3 PUFA	Placebo	ADHD-IV inattention subscore	No significant difference; study underpowered
Elsadek et al., 2021 [21]	Double-blind RCT	60	Not stated	Intractable epilepsy with ADHD	Omega-3 + risperidone + AEDs	Placebo + risperidone + AEDs	Seizure frequency, ADHD (indirect)	Significant reduction in seizure frequency
Wang et al., 2023 [22]	RCT	70	Not stated	Children with epilepsy	Comprehensive nursing program	Active control group	Anxiety and depression scores	Lower anxiety/depression scores in the intervention group at follow-up
Tutar Güven et al., 2020 [23]	RCT	Not stated	9–18 years	Youth with epilepsy	WEEP (web-based epilepsy education)	No additional education	Knowledge, self-efficacy, e-health literacy, attitudes, anxiety	Improved knowledge, self-efficacy, and literacy in both youth and parents

Quality Assessment

The quality assessment of the included studies is presented in Table 2, using appropriate tools tailored to each study design. Most randomized controlled trials were assessed using the RoB 2.0 tool and were found to have a low overall risk of bias, including studies evaluating the Mental Health Intervention for Children with Epilepsy (MICE) intervention, nurse-led CBT, and omega-3 supplementation. One study, however, exhibited some concerns related to the randomization process and outcome measurement. Mixed-methods and qualitative studies were appraised using the MMAT and CASP checklists, respectively, both indicating a low risk of bias, although one study noted some concerns regarding missing data. Single-arm and before-after trials were evaluated using the National Institutes of Health (NIH) quality assessment tools and revealed some methodological limitations, particularly in areas such as missing outcome data and reporting bias. Overall, the majority of studies were methodologically sound and provided reliable evidence, though a few presented minor risks that were carefully considered in the synthesis of findings.

**Table 2:** Quality assessment of included clinical trials using RoB 2.0, MMAT, CASP, and NIH tools. RoB 2.0: Cochrane Risk of Bias 2.0 Tool; MMAT: Mixed Methods Appraisal Tool; CASP: Critical Appraisal Skills Programme; NIH: National Institutes of Health; RCT: randomized controlled trial

Study (Author, Year)	Study Design	Tool Used	Randomization Process	Deviations from Intended Interventions	Missing Outcome Data	Measurement of the Outcome	Selection of the Reported Result	Overall Risk of Bias
Bennett et al., 2024 [14]	Multicentre RCT	RoB 2.0	Low	Low	Low	Low	Low	Low
Nizza et al., 2024 [15]	Mixed-methods (within RCT)	MMAT	Low	Low	Some concerns	Low	Low	Low
Smith et al., 2025 [16]	Qualitative Study	CASP	Not applicable	Not applicable	Low	Low	Low	Low
Wu et al., 2024 [17]	RCT	RoB 2.0	Low	Low	Low	Low	Low	Low
Svanström et al., 2023 [18]	Single-arm pilot trial	NIH Pre-Post Tool	Not applicable	Low	Some concerns	Low	Some concerns	Some concerns
Thompson et al., 2020 [19]	Open-label clinical trial	NIH Before-After Tool	Not applicable	Some concerns	Low	Low	Low	Some concerns
Rheims et al., 2024 [20]	RCT	RoB 2.0	Low	Low	Low	Low	Low	Low
Elsadek et al., 2021 [21]	Double-blind RCT	RoB 2.0	Low	Low	Low	Low	Low	Low
Wang et al., 2023 [22]	RCT	RoB 2.0	Some concerns	Low	Low	Some concerns	Low	Some concerns
Tutar Güven et al., 2020 [23]	RCT	RoB 2.0	Low	Low	Low	Low	Low	Low

Discussion

This systematic review aimed to evaluate the effectiveness of various treatment approaches in addressing psychiatric symptoms and cognitive impairments among children and adolescents with epilepsy. Across the 10 included studies, a consistent trend was observed favoring psychological and educational interventions in improving mental health outcomes. MICE, evaluated in multiple studies, was particularly effective in reducing emotional and behavioral difficulties, as evidenced by improvements in Strengths and Difficulties Questionnaire (SDQ) scores, emotional regulation, and parent-child dynamics. Nurse-led CBT for parents and comprehensive nursing programs also demonstrated significant reductions in parental anxiety and depression, positively impacting child outcomes. Additionally, psychoeducational approaches like the Supporting Attention in Children with Epilepsy (SPACE) program [18] and the Web-based Epilepsy Education Program (WEEP) [23] web-based platform enhanced executive functioning, health-related quality of life (HRQOL), and e-health literacy. While cannabidiol (CBD) showed no adverse cognitive effects and omega-3 supplementation reduced seizure frequency in comorbid ADHD cases, the evidence for cognitive or psychiatric improvement from pharmacologic treatments was less robust. Overall, non-pharmacological, family-centered interventions emerged as particularly beneficial in improving both psychological well-being and functional outcomes in pediatric epilepsy.

The findings of this review have significant implications for clinical practice, particularly in emphasizing the need for integrated psychosocial interventions within pediatric epilepsy care [24]. Interventions such as the MICE consistently demonstrated substantial improvements in emotional and behavioral outcomes, as reflected in reduced SDQ scores, improved emotional regulation, and enhanced parental insight. Similarly, nurse-led CBT programs effectively alleviated parental anxiety and depression, suggesting indirect yet meaningful benefits for the psychological stability of children. Psychoeducational programs like SPACE [18] were successful in improving executive functioning and HRQOL, especially among children with attention difficulties. Notably, these interventions were not only effective across a broad pediatric population but also showed promising results in subgroups with comorbid neurodevelopmental disorders such as ASD and ID, reinforcing the value of tailored and family-centered care models in epilepsy management.

Compared to previous reviews that often focused narrowly on seizure control or isolated behavioral interventions, this review offers a more comprehensive synthesis by concurrently evaluating cognitive and psychiatric treatment strategies in pediatric epilepsy. Existing literature frequently lacks integration of psychosocial outcomes or limits its scope to pharmacological studies. In contrast, this review expands the field by including diverse interventions, ranging from psychotherapy to web-based education, thereby providing a more holistic understanding of patient-centered care. This broader scope is novel and valuable, particularly given the complex interplay between epilepsy and mental health in children. The consistent demonstration of benefits from psychological and educational interventions aligns with growing evidence favoring multimodal care but also highlights the need for future reviews and trials to adopt similarly integrative frameworks [25,26].

The therapeutic benefits observed in this review can be understood through several neurodevelopmental and psychological mechanisms. Interventions like CBT and psychoeducation likely function by enhancing coping strategies, emotional regulation, and parental competence, thereby buffering the child against chronic stress and reinforcing adaptive behaviors. Psychoeducational programs also improve health literacy and self-management, fostering a greater sense of control and engagement among both children and their caregivers. Nutritional interventions, such as omega-3 supplementation, may exert neuroprotective and anti-inflammatory effects that support neural function and plasticity [27]. Importantly, the relationship between epilepsy and psychiatric/cognitive symptoms is bidirectional: seizures and antiepileptic drugs may impair mood and cognition, while stress and emotional dysregulation can, in turn, exacerbate seizure activity. This dynamic underscores the need for interventions that address not only the neurological but also the psychosocial dimensions of epilepsy.

This review is strengthened by a comprehensive and systematic literature search adhering to the PRISMA guidelines, ensuring methodological rigor and transparency. A notable advantage is the inclusion of both pharmacological and non-pharmacological interventions, enabling a holistic understanding of treatment strategies for psychiatric and cognitive symptoms in children with epilepsy. The exclusive focus on the pediatric population further enhances its relevance for clinical application in child neurology and psychiatry. However, several limitations must be acknowledged. Some included studies had small sample sizes or employed single-arm designs without control groups, limiting the strength of causal inferences. Additionally, methodological heterogeneity was evident across studies in terms of outcome measures, assessment tools, and population characteristics, making direct comparisons challenging. A few trials, particularly open-label or pilot studies, were susceptible to performance and detection bias due to a lack of blinding. While most studies were of moderate to high quality, some had an unclear or only partially reported risk of bias, necessitating cautious interpretation of findings.

Despite promising evidence, important gaps persist in the literature regarding the treatment of psychiatric and cognitive impairments in pediatric epilepsy. Long-term outcomes remain underexplored, with most studies assessing short-term or immediate post-intervention effects. There is a critical lack of interventions tailored specifically to children with comorbid conditions such as ASD, ID, or ADHD, who may respond differently to standard therapies. Additionally, certain studies, like the Rheims et al. [20] trial on polyunsaturated fatty acids (PUFA) supplementation, were underpowered due to early termination or small sample sizes, limiting the reliability of their conclusions. Variability and lack of standardization in cognitive outcome measures across studies also reduce the comparability and reproducibility of findings. These gaps underscore the need for more targeted, inclusive, and methodologically rigorous research in this field.

Future research should prioritize large-scale, multicenter randomized controlled trials with longer follow-up durations to assess the sustainability of treatment benefits and potential long-term impacts on neurodevelopment. Integrating routine mental health screening into pediatric epilepsy care pathways could aid in early identification and timely intervention for psychiatric symptoms, improving overall disease management. Additionally, there is a need to standardize outcome measures related to psychiatric and cognitive function, such as using validated and consistent tools like the SDQ or NIH Toolbox Cognition Battery (NIHTB-CB) [28], to facilitate comparison across studies and enable future meta-analyses. Developing tailored interventions for children with epilepsy and neurodevelopmental comorbidities and evaluating the effectiveness of digital or community-based delivery models could further enhance the accessibility and scalability of care.

## Conclusions

In conclusion, this systematic review highlights the critical importance of addressing psychiatric symptoms and cognitive impairments as integral components of comprehensive epilepsy care in children. The evidence demonstrates that psychological and educational interventions, particularly modular therapies like MICE, nurse-led cognitive behavioral programs, and psychoeducational models such as SPACE and WEEP, are not only feasible but also effective in improving emotional well-being, behavioral functioning, and cognitive outcomes in pediatric epilepsy populations. These approaches offer meaningful benefits beyond seizure control, emphasizing the need for a multidimensional treatment paradigm that considers the child’s mental health and developmental needs. The findings underscore the value of integrating family-centered, accessible, and evidence-based psychosocial support into standard epilepsy management. By focusing on both neurocognitive and psychiatric outcomes, this review contributes a vital perspective to the evolving landscape of pediatric neurology, advocating for a shift toward more holistic, individualized, and quality-of-life-oriented care models for children living with epilepsy.
